# Ultrafast and memory-efficient alignment of short DNA sequences to the human genome

**DOI:** 10.1186/gb-2009-10-3-r25

**Published:** 2009-03-04

**Authors:** Ben Langmead, Cole Trapnell, Mihai Pop, Steven L Salzberg

**Affiliations:** 1 Center for Bioinformatics and Computational Biology, Institute for Advanced Computer Studies, University of Maryland, College Park, MD 20742, USA

## Abstract

Bowtie is an ultrafast, memory-efficient alignment program for aligning short DNA sequence reads to large genomes. For the human genome, Burrows-Wheeler indexing allows Bowtie to align more than 25 million reads per CPU hour with a memory footprint of approximately 1.3 gigabytes. Bowtie extends previous Burrows-Wheeler techniques with a novel quality-aware backtracking algorithm that permits mismatches. Multiple processor cores can be used simultaneously to achieve even greater alignment speeds. Bowtie is open source .

## Abstract 2

Bowtie: a new ultrafast memory-efficient tool for the alignment of short DNA sequence reads to large genomes.

## Rationale

Improvements in the efficiency of DNA sequencing have both broadened the applications for sequencing and dramatically increased the size of sequencing datasets. Technologies from Illumina (San Diego, CA, USA) and Applied Biosystems (Foster City, CA, USA) have been used to profile methylation patterns (MeDIP-Seq) [1], to map DNA-protein interactions (ChIP-Seq) [2], and to identify differentially expressed genes (RNA-Seq) [3] in the human genome and other species. The Illumina instrument was recently used to re-sequence three human genomes, one from a cancer patient and two from previously unsequenced ethnic groups [4-6]. Each of these studies required the alignment of large numbers of short DNA sequences ('short reads') onto the human genome. For example, two of the studies [4,5] used the short read alignment tool Maq [7] to align more than 130 billion bases (about 45× coverage) of short Illumina reads to a human reference genome in order to detect genetic variations. The third human re-sequencing study [6] used the SOAP program [8] to align more than 100 billion bases to the reference genome. In addition to these projects, the 1,000 Genomes project is in the process of using high-throughput sequencing instruments to sequence a total of about six trillion base pairs of human DNA [9].

With existing methods, the computational cost of aligning many short reads to a mammalian genome is very large. For example, extrapolating from the results presented here in Tables 1 and 2, one can see that Maq would require more than 5 central processing unit (CPU)-months and SOAP more than 3 CPU-years to align the 140 billion bases from the study by Ley and coworkers [5]. Although using Maq or SOAP for this purpose has been shown to be feasible by using multiple CPUs, there is a clear need for new tools that consume less time and computational resources.

**Table 1:** Bowtie alignment performance versus SOAP and Maq

	Platform	CPU time	Wall clock time	Reads mapped per hour (millions)	Peak virtual memory footprint (megabytes)	Bowtie speed-up	Reads aligned (%)
Bowtie -v 2	Server	15 m 7 s	15 m 41 s	33.8	1,149	-	67.4
SOAP		91 h 57 m 35 s	91 h 47 m 46 s	0.10	13,619	351×	67.3

Bowtie	PC	16 m 41 s	17 m 57 s	29.5	1,353	-	71.9
Maq		17 h 46 m 35 s	17 h 53 m 7 s	0.49	804	59.8×	74.7

Bowtie	Server	17 m 58 s	18 m 26 s	28.8	1,353	-	71.9
Maq		32 h 56 m 53 s	32 h 58 m 39 s	0.27	804	107×	74.7

The performance and sensitivity of Bowtie v0.9.6, SOAP v1.10, and Maq v0.6.6 when aligning 8.84 M reads from the 1,000 Genome project (National Center for Biotechnology Information Short Read Archive: SRR001115) trimmed to 35 base pairs. The 'soap.contig' version of the SOAP binary was used. SOAP could not be run on the PC because SOAP's memory footprint exceeds the PC's physical memory. For the SOAP comparison, Bowtie was invoked with '-v 2' to mimic SOAP's default matching policy (which allows up to two mismatches in the alignment and disregards quality values). For the Maq comparison Bowtie is run with its default policy, which mimics Maq's default policy of allowing up to two mismatches during the first 28 bases and enforcing an overall limit of 70 on the sum of the quality values at all mismatched positions. To make Bowtie's memory footprint more comparable to Maq's, Bowtie is invoked with the '-z' option in all experiments to ensure only the forward or mirror index is resident in memory at one time. CPU, central processing unit.

**Table 2:** Bowtie alignment performance versus Maq with filtered read set

	Platform	CPU time	Wall clock time	Reads mapped per hour (millions)	Peak virtual memory footprint (megabytes)	Bowtie speed up	Reads aligned (%)
Bowtie	PC	16 m 39 s	17 m 47 s	29.8	1,353	-	74.9
Maq		11 h 15 m 58 s	11 h 22 m 2 s	0.78	804	38.4×	78.0

Bowtie	Server	18 m 20 s	18 m 46 s	28.3	1,352	-	74.9
Maq		18 h 49 m 7 s	18 h 50 m 16 s	0.47	804	60.2×	78.0

Performance and sensitivity of Bowtie v0.9.6 and Maq v0.6.6 when the read set is filtered using Maq's 'catfilter' command to eliminate poly-A artifacts. The filter eliminates 438,145 out of 8,839,010 reads. Other experimental parameters are identical to those of the experiments in Table 1. CPU, central processing unit.

Maq and SOAP take the same basic algorithmic approach as other recent read mapping tools such as RMAP [10], ZOOM [11], and SHRiMP [12]. Each tool builds a hash table of short oligomers present in either the reads (SHRiMP, Maq, RMAP, and ZOOM) or the reference (SOAP). Some employ recent theoretical advances to align reads quickly without sacrificing sensitivity. For example, ZOOM uses 'spaced seeds' to significantly outperform RMAP, which is based on a simpler algorithm developed by Baeza-Yaetes and Perleberg [13]. Spaced seeds have been shown to yield higher sensitivity than contiguous seeds of the same length [14,15]. SHRiMP employs a combination of spaced seeds and the Smith-Waterman [16] algorithm to align reads with high sensitivity at the expense of speed. Eland is a commercial alignment program available from Illumina that uses a hash-based algorithm to align reads.

Bowtie uses a different and novel indexing strategy to create an ultrafast, memory-efficient short read aligner geared toward mammalian re-sequencing. In our experiments using reads from the 1,000 Genomes project, Bowtie aligns 35-base pair (bp) reads at a rate of more than 25 million reads per CPU-hour, which is more than 35 times faster than Maq and 300 times faster than SOAP under the same conditions (see Tables 1 and 2). Bowtie employs a Burrows-Wheeler index based on the full-text minute-space (FM) index, which has a memory footprint of only about 1.3 gigabytes (GB) for the human genome. The small footprint allows Bowtie to run on a typical desktop computer with 2 GB of RAM. The index is small enough to be distributed over the internet and to be stored on disk and re-used. Multiple processor cores can be used simultaneously to achieve even greater alignment speed. We have used Bowtie to align 14.3× coverage worth of human Illumina reads from the 1,000 Genomes project in about 14 hours on a single desktop computer with four processor cores.

Bowtie makes a number of compromises to achieve this speed, but these trade-offs are reasonable within the context of mammalian re-sequencing projects. If one or more exact matches exist for a read, then Bowtie is guaranteed to report one, but if the best match is an inexact one then Bowtie is not guaranteed in all cases to find the highest quality alignment. With its highest performance settings, Bowtie may fail to align a small number of reads with valid alignments, if those reads have multiple mismatches. If the stronger guarantees are desired, Bowtie supports options that increase accuracy at the cost of some performance. For instance, the '--best' option will guarantee that all alignments reported are best in terms of minimizing mismatches in the seed portion of the read, although this option incurs additional computational cost.

With its default options, Bowtie's sensitivity measured in terms of reads aligned is equal to SOAP's and somewhat less than Maq's. Command line options allow the user to increase sensitivity at the cost of greater running time, and to enable Bowtie to report multiple hits for a read. Bowtie can align reads as short as four bases and as long as 1,024 bases. The input to a single run of Bowtie may comprise a mixture of reads with different lengths.

## Bowtie description and results

Bowtie indexes the reference genome using a scheme based on the Burrows-Wheeler transform (BWT) [17] and the FM index [18,19]. A Bowtie index for the human genome fits in 2.2 GB on disk and has a memory footprint of as little as 1.3 GB at alignment time, allowing it to be queried on a workstation with under 2 GB of RAM.

The common method for searching in an FM index is the exact-matching algorithm of Ferragina and Manzini [18]. Bowtie does not simply adopt this algorithm because exact matching does not allow for sequencing errors or genetic variations. We introduce two novel extensions that make the technique applicable to short read alignment: a quality-aware backtracking algorithm that allows mismatches and favors high-quality alignments; and 'double indexing', a strategy to avoid excessive backtracking. The Bowtie aligner follows a policy similar to Maq's, in that it allows a small number of mismatches within the high-quality end of each read, and it places an upper limit on the sum of the quality values at mismatched alignment positions.

### Burrows-Wheeler indexing

The BWT is a reversible permutation of the characters in a text. Although originally developed within the context of data compression, BWT-based indexing allows large texts to be searched efficiently in a small memory footprint. It has been applied to bioinformatics applications, including oligomer counting [20], whole-genome alignment [21], tiling microarray probe design [22], and Smith-Waterman alignment to a human-sized reference [23].

**Figure 1:**
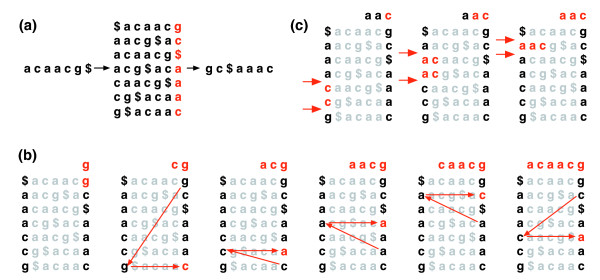
Burrows-Wheeler transform. **(a) **The Burrows-Wheeler matrix and transformation for 'acaacg'. **(b) **Steps taken by EXACTMATCH to identify the range of rows, and thus the set of reference suffixes, prefixed by 'aac'. **(c) **UNPERMUTE repeatedly applies the last first (LF) mapping to recover the original text (in red on the top line) from the Burrows-Wheeler transform (in black in the rightmost column).

The Burrows-Wheeler transformation of a text T, BWT(T), is constructed as follows. The character $ is appended to T, where $ is not in T and is lexicographically less than all characters in T. The Burrows-Wheeler matrix of T is constructed as the matrix whose rows comprise all cyclic rotations of T$. The rows are then sorted lexicographically. BWT(T) is the sequence of characters in the rightmost column of the Burrows-Wheeler matrix (Figure 1a). BWT(T) has the same length as the original text T.

This matrix has a property called 'last first (LF) mapping'. The i^th ^occurrence of character X in the last column corresponds to the same text character as the i^th ^occurrence of X in the first column. This property underlies algorithms that use the BWT index to navigate or search the text. Figure 1b illustrates UNPERMUTE, an algorithm that applies the LF mapping repeatedly to re-create T from BWT(T).

The LF mapping is also used in exact matching. Because the matrix is sorted lexicographically, rows beginning with a given sequence appear consecutively. In a series of steps, the EXACTMATCH algorithm (Figure 1c) calculates the range of matrix rows beginning with successively longer suffixes of the query. At each step, the size of the range either shrinks or remains the same. When the algorithm completes, rows beginning with S_0 _(the entire query) correspond to exact occurrences of the query in the text. If the range is empty, the text does not contain the query. UNPERMUTE is attributable to Burrows and Wheeler [17] and EXACTMATCH to Ferragina and Manzini [18]. See Additional data file 1 (Supplementary Discussion 1) for details.

### Searching for inexact alignments

EXACTMATCH is insufficient for short read alignment because alignments may contain mismatches, which may be due to sequencing errors, genuine differences between reference and query organisms, or both. We introduce an alignment algorithm that conducts a backtracking search to quickly find alignments that satisfy a specified alignment policy. Each character in a read has a numeric quality value, with lower values indicating a higher likelihood of a sequencing error. Our alignment policy allows a limited number of mismatches and prefers alignments where the sum of the quality values at all mismatched positions is low.

The search proceeds similarly to EXACTMATCH, calculating matrix ranges for successively longer query suffixes. If the range becomes empty (a suffix does not occur in the text), then the algorithm may select an already-matched query position and substitute a different base there, introducing a mismatch into the alignment. The EXACTMATCH search resumes from just after the substituted position. The algorithm selects only those substitutions that are consistent with the alignment policy and which yield a modified suffix that occurs at least once in the text. If there are multiple candidate substitution positions, then the algorithm greedily selects a position with a minimal quality value.

**Figure 2:**
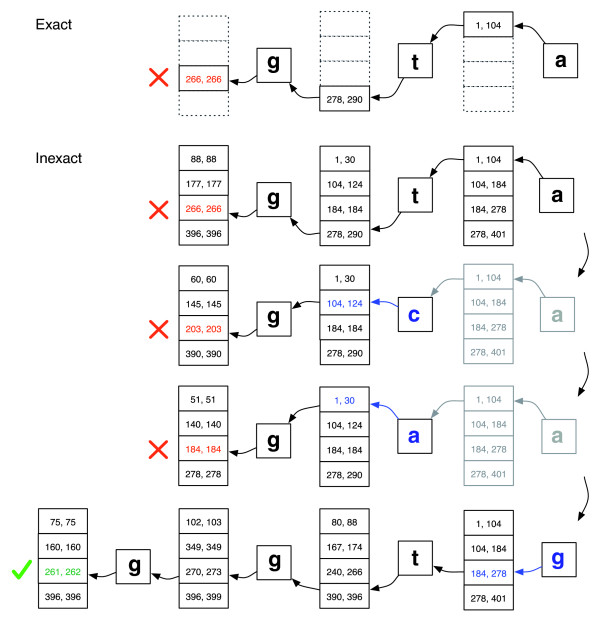
Exact matching versus inexact alignment. Illustration of how EXACTMATCH (top) and Bowtie's aligner (bottom) proceed when there is no exact match for query 'ggta' but there is a one-mismatch alignment when 'a' is replaced by 'g'. Boxed pairs of numbers denote ranges of matrix rows beginning with the suffix observed up to that point. A red X marks where the algorithm encounters an empty range and either aborts (as in EXACTMATCH) or backtracks (as in the inexact algorithm). A green check marks where the algorithm finds a nonempty range delimiting one or more occurrences of a reportable alignment for the query.

Backtracking scenarios play out within the context of a stack structure that grows when a new substitution is introduced and shrinks when the aligner rejects all candidate alignments for the substitutions currently on the stack. See Figure 2 for an illustration of how the search might proceed.

In short, Bowtie conducts a quality-aware, greedy, randomized, depth-first search through the space of possible alignments. If a valid alignment exists, then Bowtie will find it (subject to the backtrack ceiling discussed in the following section). Because the search is greedy, the first valid alignment encountered by Bowtie will not necessarily be the 'best' in terms of number of mismatches or in terms of quality. The user may instruct Bowtie to continue searching until it can prove that any alignment it reports is 'best' in terms of number of mismatches (using the option --best). In our experience, this mode is two to three times slower than the default mode. We expect that the faster default mode will be preferred for large re-sequencing projects.

The user may also opt for Bowtie to report all alignments up to a specified number (option -k) or all alignments with no limit on the number (option -a) for a given read. If in the course of its search Bowtie finds N possible alignments for a given set of substitutions, but the user has requested only K alignments where K < N, Bowtie will report K of the N alignments selected at random. Note that these modes can be much slower than the default. In our experience, for example, -k 1 is more than twice as fast as -k 2.

### Excessive backtracking

The aligner as described so far can, in some cases, encounter sequences that cause excessive backtracking. This occurs when the aligner spends most of its effort fruitlessly backtracking to positions close to the 3' end of the query. Bowtie mitigates excessive backtracking with the novel technique of 'double indexing'. Two indices of the genome are created: one containing the BWT of the genome, called the 'forward' index, and a second containing the BWT of the genome with its character sequence reversed (not reverse complemented) called the 'mirror' index. To see how this helps, consider a matching policy that allows one mismatch in the alignment. A valid alignment with one mismatch falls into one of two cases according to which half of the read contains the mismatch. Bowtie proceeds in two phases corresponding to those two cases. Phase 1 loads the forward index into memory and invokes the aligner with the constraint that it may not substitute at positions in the query's right half. Phase 2 uses the mirror index and invokes the aligner on the reversed query, with the constraint that the aligner may not substitute at positions in the reversed query's right half (the original query's left half). The constraints on backtracking into the right half prevent excessive backtracking, whereas the use of two phases and two indices maintains full sensitivity.

Unfortunately, it is not possible to avoid excessive backtracking fully when alignments are permitted to have two or more mismatches. In our experiments, we have observed that excessive backtracking is significant only when a read has many low-quality positions and does not align or aligns poorly to the reference. These cases can trigger in excess of 200 backtracks per read because there are many legal combinations of low-quality positions to be explored before all possibilities are exhausted. We mitigate this cost by enforcing a limit on the number of backtracks allowed before a search is terminated (default: 125). The limit prevents some legitimate, low-quality alignments from being reported, but we expect that this is a desirable trade-off for most applications.

### Phased Maq-like search

Bowtie allows the user to select the number of mismatches permitted (default: two) in the high-quality end of a read (default: the first 28 bases) as well as the maximum acceptable quality distance of the overall alignment (default: 70). Quality values are assumed to follow the definition in PHRED [24], where p is the probability of error and Q = -10log p.

Both the read and its reverse complement are candidates for alignment to the reference. For clarity, this discussion considers only the forward orientation. See Additional data file 1 (Supplementary Discussion 2) for an explanation of how the reverse complement is incorporated.

The first 28 bases on the high-quality end of the read are termed the 'seed'. The seed consists of two halves: the 14 bp on the high-quality end (usually the 5' end) and the 14 bp on the low-quality end, termed the 'hi-half' and the 'lo-half', respectively. Assuming the default policy (two mismatches permitted in the seed), a reportable alignment will fall into one of four cases: no mismatches in seed (case 1); no mismatches in hi-half, one or two mismatches in lo-half (case 2); no mismatches in lo-half, one or two mismatches in hi-half (case 3); and one mismatch in hi-half, one mismatch in lo-half (case 4).

All cases allow any number of mismatches in the nonseed part of the read and all cases are also subject to the quality distance constraint.

**Figure 3:**
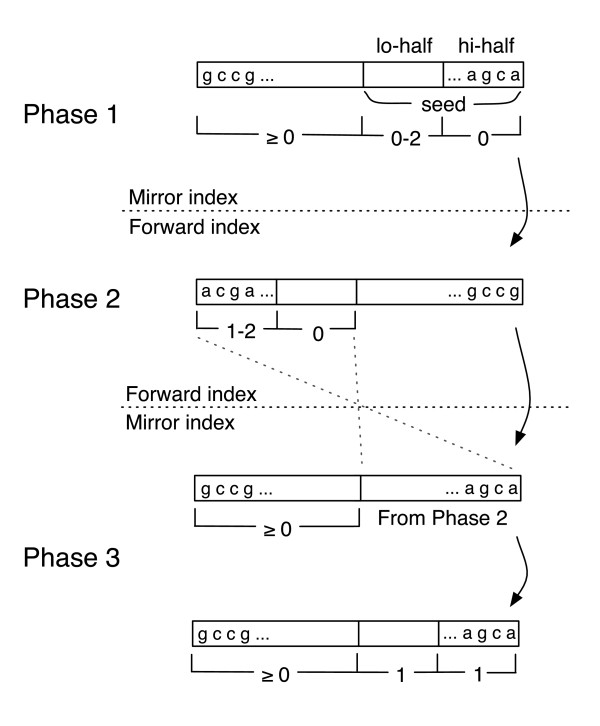
The three phases of the Bowtie algorithm for the Maq-like policy. A three-phase approach finds alignments for two-mismatch cases 1 to 4 while minimizing backtracking. Phase 1 uses the mirror index and invokes the aligner to find alignments for cases 1 and 2. Phases 2 and 3 cooperate to find alignments for case 3: Phase 2 finds partial alignments with mismatches only in the hi-half, and phase 3 attempts to extend those partial alignments into full alignments. Finally, phase 3 invokes the aligner to find alignments for case 4.

The Bowtie algorithm consists of three phases that alternate between using the forward and mirror indices, as illustrated in Figure 3. Phase 1 uses the mirror index and invokes the aligner to find alignments for cases 1 and 2. Phases 2 and 3 cooperate to find alignments for case 3: Phase 2 finds partial alignments with mismatches only in the hi-half and phase 3 attempts to extend those partial alignments into full alignments. Finally, phase 3 invokes the aligner to find alignments for case 4.

### Performance results

We evaluated the performance of Bowtie using reads from the 1,000 Genomes project pilot (National Center for Biotechnology Information [NCBI] Short Read Archive:SRR001115). A total of 8.84 million reads, about one lane of data from an Illumina instrument, were trimmed to 35 bp and aligned to the human reference genome [NCBI build 36.3]. Unless specified otherwise, read data are not filtered or modified (besides trimming) from how they appear in the archive. This leads to about 70% to 75% of reads aligning somewhere to the genome. In our experience, this is typical for raw data from the archive. More aggressive filtering leads to higher alignment rates and faster alignment.

All runs were performed on a single CPU. Bowtie speedups were calculated as a ratio of wall-clock alignment times. Both wall-clock and CPU times are given to demonstrate that input/output load and CPU contention are not significant factors.

The time required to build the Bowtie index was not included in the Bowtie running times. Unlike competing tools, Bowtie can reuse a pre-computed index for the reference genome across many alignment runs. We anticipate most users will simply download such indices from a public repository. The Bowtie site [25] provides indices for current builds of the human, chimp, mouse, dog, rat, and *Arabidopsis thaliana *genomes, as well as many others.

Results were obtained on two hardware platforms: a desktop workstation with 2.4 GHz Intel Core 2 processor and 2 GB of RAM; and a large-memory server with a four-core 2.4 GHz AMD Opteron processor and 32 GB of RAM. These are denoted 'PC' and 'server', respectively. Both PC and server run Red Hat Enterprise Linux AS release 4.

### Comparison to SOAP and Maq

Maq is a popular aligner [1,4,5,26,27] that is among the fastest competing open source tools for aligning millions of Illumina reads to the human genome. SOAP is another open source tool that has been reported and used in short-read projects [6,28].

Table 1 presents the performance and sensitivity of Bowtie v0.9.6, SOAP v1.10, and Maq v0.6.6. SOAP could not be run on the PC because SOAP's memory footprint exceeds the PC's physical memory. The 'soap.contig' version of the SOAP binary was used. For comparison with SOAP, Bowtie was invoked with '-v 2' to mimic SOAP's default matching policy (which allows up to two mismatches in the alignment and disregards quality values), and with '--maxns 5' to simulate SOAP's default policy of filtering out reads with five or more no-confidence bases. For the Maq comparison Bowtie is run with its default policy, which mimics Maq's default policy of allowing up to two mismatches in the first 28 bases and enforcing an overall limit of 70 on the sum of the quality values at all mismatched read positions. To make Bowtie's memory footprint more comparable to Maq's, Bowtie is invoked with the '-z' option in all experiments to ensure that only the forward or mirror index is resident in memory at one time.

The number of reads aligned indicates that SOAP (67.3%) and Bowtie -v 2 (67.4%) have comparable sensitivity. Of the reads aligned by either SOAP or Bowtie, 99.7% were aligned by both, 0.2% were aligned by Bowtie but not SOAP, and 0.1% were aligned by SOAP but not Bowtie. Maq (74.7%) and Bowtie (71.9%) also have roughly comparable sensitivity, although Bowtie lags by 2.8%. Of the reads aligned by either Maq or Bowtie, 96.0% were aligned by both, 0.1% were aligned by Bowtie but not Maq, and 3.9% were aligned by Maq but not Bowtie. Of the reads mapped by Maq but not Bowtie, almost all are due to a flexibility in Maq's alignment algorithm that allows some alignments to have three mismatches in the seed. The remainder of the reads mapped by Maq but not Bowtie are due to Bowtie's backtracking ceiling.

Maq's documentation mentions that reads containing 'poly-A artifacts' can impair Maq's performance. Table 2 presents performance and sensitivity of Bowtie and Maq when the read set is filtered using Maq's 'catfilter' command to eliminate poly-A artifacts. The filter eliminates 438,145 out of 8,839,010 reads. Other experimental parameters are identical to those of the experiments in Table 1, and the same observations about the relative sensitivity of Bowtie and Maq apply here.

### Read length and performance

As sequencing technology improves, read lengths are growing beyond the 30-bp to 50-bp commonly seen in public databases today. Bowtie, Maq, and SOAP support reads of lengths up to 1,024, 63, and 60 bp, respectively, and Maq versions 0.7.0 and later support read lengths up to 127 bp. Table 3 shows performance results when the three tools are each used to align three sets of 2 M untrimmed reads, a 36-bp set, a 50-bp set and a 76-bp set, to the human genome on the server platform. Each set of 2 M is randomly sampled from a larger set (NCBI Short Read Archive: SRR003084 for 36-bp, SRR003092 for 50-bp, SRR003196 for 76-bp). Reads were sampled such that the three sets of 2 M have uniform per-base error rate, as calculated from per-base Phred qualities. All reads pass through Maq's 'catfilter'.

**Table 3:** Varying read length using Bowtie, Maq and SOAP

Length	Program	CPU time	Wall clock time	Peak virtual memory footprint (megabytes)	Bowtie speed-up	Reads aligned (%)
36 bp	Bowtie	6 m 15 s	6 m 21 s	1,305	-	62.2
	
	Maq	3 h 52 m 26 s	3 h 52 m 54 s	804	36.7×	65.0
	
	Bowtie -v 2	4 m 55 s	5 m 00 s	1,138	-	55.0
	
	SOAP	16 h 44 m 3 s	18 h 1 m 38 s	13,619	216×	55.1

50 bp	Bowtie	7 m 11 s	7 m 20 s	1,310	-	67.5
	
	Maq	2 h 39 m 56 s	2 h 40 m 9 s	804	21.8×	67.9
	
	Bowtie -v 2	5 m 32 s	5 m 46 s	1,138	-	56.2
	
	SOAP	48 h 42 m 4 s	66 h 26 m 53 s	13,619	691×	56.2

76 bp	Bowtie	18 m 58 s	19 m 6 s	1,323	-	44.5
	
	Maq 0.7.1	4 h 45 m 7 s	4 h 45 m 17 s	1,155	14.9×	44.9
	
	Bowtie -v 2	7 m 35 s	7 m 40 s	1,138	-	31.7

The performance of Bowtie v0.9.6, SOAP v1.10, and Maq versions v0.6.6 and v0.7.1 on the server platform when aligning 2 M untrimmed reads from the 1,000 Genome project (National Center for Biotechnology Information Short Read Archive: SRR003084 for 36 base pairs [bp], SRR003092 for 50 bp, and SRR003196 for 76 bp). For each read length, the 2 M reads were randomly sampled from the FASTQ file downloaded from the Archive such that the average per-base error rate as measured by quality values was uniform across the three sets. All reads pass through Maq's "catfilter". Maq v0.7.1 was used for the 76-bp reads because v0.6.6 does not support reads longer than 63 bp. SOAP is excluded from the 76-bp experiment because it does not support reads longer than 60 bp. Other experimental parameters are identical to those of the experiments in Table 1. CPU, central processing unit.

Bowtie is run both in its Maq-like default mode and in its SOAP-like '-v 2' mode. Bowtie is also given the '-z' option to ensure that only the forward or mirror index is resident in memory at one time. Maq v0.7.1 was used instead of Maq v0.6.6 for the 76-bp set because v0.6.6 cannot align reads longer than 63 bp. SOAP was not run on the 76-bp set because it does not support reads longer than 60 bp.

The results show that Maq's algorithm scales better overall to longer read lengths than Bowtie or SOAP. However, Bowtie in SOAP-like '-v 2' mode also scales very well. Bowtie in its default Maq-like mode scales well from 36-bp to 50-bp reads but is substantially slower for 76-bp reads, although it is still more than an order of magnitude faster than Maq.

### Parallel performance

Alignment can be parallelized by distributing reads across concurrent search threads. Bowtie allows the user to specify a desired number of threads (option -p); Bowtie then launches the specified number of threads using the pthreads library. Bowtie threads synchronize with each other when fetching reads, outputting results, switching between indices, and performing various forms of global bookkeeping, such as marking a read as 'done'. Otherwise, threads are free to operate in parallel, substantially speeding up alignment on computers with multiple processor cores. The memory image of the index is shared by all threads, and so the footprint does not increase substantially when multiple threads are used. Table 4 shows performance results for running Bowtie v0.9.6 on the four-core server with one, two, and four threads.

**Table 4:** Bowtie parallel alignment performance

	CPU time	Wall clock time	Reads mapped per hour (millions)	Peak virtual memory footprint (megabytes)	Speedup
Bowtie, one thread	18 m 19 s	18 m 46 s	28.3	1,353	-
Bowtie, two threads	20 m 34 s	10 m 35 s	50.1	1,363	1.77×
Bowtie, four threads	23 m 9 s	6 m 1 s	88.1	1,384	3.12×

Performance results for running Bowtie v0.9.6 on the four-core server with one, two, and four threads. Other experimental parameters are identical to those of the experiments in Table 1. CPU, central processing unit.

### Index building

Bowtie uses a flexible indexing algorithm [29] that can be configured to trade off between memory usage and running time. Table 5 illustrates this trade-off when indexing the entire human reference genome (NCBI build 36.3, contigs). Runs were performed on the server platform. The indexer was run four times with different upper limits on memory usage.

**Table 5:** Bowtie index building performance

Physical memory target (GB)	Actual peak memory footprint (GB)	Wall clock time
16	14.4	4 h 36 m
8	5.84	5 h 5 m
4	3.39	7 h 40 m
2	1.39	21 h 30 m

Performance results and memory footprints of running the Bowtie v0.9.6 indexer on the whole human genome (National Center for Biotechnology Information build 36.3, contigs). Runs were performed on the server platform. The indexer was run four times with different upper limits on memory usage. See Additional data file 1 (Supplementary Discussion 3 and Supplementary Table 1) for details.

The reported times compare favorably with alignment times of competing tools that perform indexing during alignment. Less than 5 hours is required for Bowtie to both build and query a whole-human index with 8.84 million reads from the 1,000 Genome project (NCBI Short Read Archive:SRR001115) on a server, more than sixfold faster than the equivalent Maq run. The bottom-most row illustrates that the Bowtie indexer, with appropriate arguments, is memory-efficient enough to run on a typical workstation with 2 GB of RAM. Additional data file 1 (Supplementary discussions 3 and 4) explains the algorithm and the contents of the resulting index.

### Software

Bowtie is written in C++ and uses the SeqAn library [30]. The converter to the Maq mapping format uses code from Maq.

## Discussion

Bowtie exhibits a large performance advantage over both Maq and SOAP when mapping reads to the human genome. Bowtie's sensitivity in terms of reads aligned is comparable to that of SOAP and slightly less than Maq's, although the user may use command-line options to trade slower running time for greater sensitivity. Unlike SOAP, Bowtie's 1.3 GB memory footprint allows it to run on a typical PC with 2 GB of RAM. Bowtie aligns Illumina reads to the human genome at a rate of over 25 million reads per hour. Multiple processor cores can run parallel Bowtie threads to achieve even greater alignment speed; experiments show a speed up of 3.12 for four threads on a typical Opteron server.

Unlike many other short-read aligners, Bowtie creates a permanent index of the reference that may be re-used across alignment runs. Building the index is fast - Bowtie outperforms competing tools when aligning lanes of Illumina reads even with index construction time included. At 2.2 GB for the human genome, the on-disk size of a Bowtie index is small enough to distribute over the internet. The Bowtie website hosts pre-built indices for the human genome and several other model organisms including chimp, dog, rat, mouse, and chicken.

Bowtie's speed and small memory footprint are due chiefly to its use of the Burrows-Wheeler index in combination with the novel, quality-aware, backtracking algorithm introduced here. Double indexing is used to avoid the performance penalty of excessive backtracking.

Bowtie supports standard FASTQ and FASTA input formats, and comes with a conversion program that allows Bowtie output to be used with Maq's consensus generator and single nucleotide polymorphism caller.

Bowtie does not yet support paired-end alignment or alignments with insertions or deletions, although both improvements are planned for the future. Paired-end alignment is not difficult to implement in Bowtie's framework, and we expect that Bowtie's performance advantage will be comparable to, though perhaps somewhat less than, that of unpaired alignment mode. Support for insertions and deletions is also a conceptually straightforward addition.

Bowtie is free, open source software available from the Bowtie website [25].

## Abbreviations

bp: base pair; BWT: Burrows-Wheeler transform; CPU: central processing unit; FM: full-text minute-space; GB: gigabytes; LF: last first; NCBI: National Center for Biotechnology Information.

## Authors' contributions

BL developed the algorithms, collected results, and wrote most of the software. CT wrote some of the software. CT and MP contributed to discussions on algorithms. BL, CT, MP, and SLS wrote the manuscript.

## Additional data files

The following additional data are included with the online version of this article: a document containing supplementary discussions, tables, and figures pertaining to algorithms for navigating the Burrows-Wheeler transform, the full four-phase version of the alignment algorithm that incorporates the reverse-complement, index construction, and components of the index (Additional data file 1).

## Supplementary Material

Additional data file 1 Presented are supplementary discussions, tables, and figures pertaining to algorithms for navigating the Burrows-Wheeler transform, the full four-phase version of the alignment algorithm that incorporates the reverse-complement, index construction, and components of the index. Click here for file
