# Supplementary material for: Ultrafast and memory-efficient alignment of short DNA sequences to the human genome
Source: Genome Biol. 2009 Mar 4;10(3):R25. doi: 10.1186/gb-2009-10-3-r25 (PMC2690996; doi:10.1186/gb-2009-10-3-r25)
Supplement: Additional data file 1 — Presented are supplementary discussions, tables, and figures pertaining to algorithms for navigating the Burrows-Wheeler transform, the full four-phase version of the alignment algorithm that incorporates the reverse-complement, index construction, and components of the index. [file gb-2009-10-3-r25-S1.doc]

**Supplementary materials**

**Supplementary Discussion 1:** BWT-navigating algorithms

**
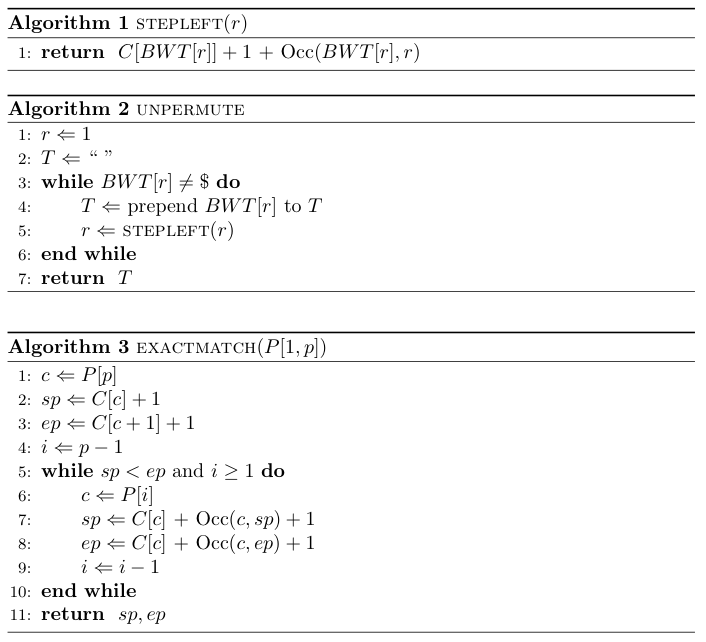
**

**Supplementary figure 1**: **Algorithms for navigating the Burrows-Wheeler transform [1].**

The pseudocode is implemented in terms of an array C[c] that maps character c to the total number of occurrences in BWT of characters lexicographically less than c, and function Occ(c, r) that calculates the number of occurrences of c in BWT up to but not including the element at index r. C[c] is easily pre-calculated.

As shown, UNPERMUTE and EXACTMATCH seem to scale poorly to large texts.  The problem is that each call to Occ examines a number of characters linear in the length of BWT(T).  Ferragina and Manzini [1] propose pre-calculating periodic character occurrence counts to reduce Occ's runtime, potentially to a constant factor. Bowtie implements a simple version of that scheme.

EXACTMATCH finds the range of matrix rows beginning with a given query string.  Each row corresponds to an exact match of the query somewhere in the text. But more work is required to determine, for a given row, which text offset it appears at. Ferragina and Manzini [1] propose a solution whereby some rows of the matrix are marked with pre-calculated text offsets.  To retrieve a row’s text offset, we first check if the row is marked.  If so, we report its pre-calculated text offset.  If not, we repeatedly invoke STEPLEFT until a marked row is reached and report its text offset plus the number of times STEPLEFT was invoked.  Marking more rows allows text offsets to be calculated faster, but marking fewer rows reduces the index size.  Bowtie adopts this scheme with a default (but configurable) policy of marking every 32nd row.

**Supplementary Discussion 2:** Full phased algorithm

The main body discusses Bowtie’s algorithm with respect to the forward-oriented read only. Incorporating the reverse complement requires introducing a new phase to the beginning of the algorithm that uses the forward index. The new phase becomes Phase 1 and the three phases described previously become Phases 2-4. The steps required to align the reverse-complement read are analogous to those of the forward-oriented read, but shifted forward by one phase. The entire process is “packed” into four phases by interleaving the processing of the forward-oriented and reverse-complement versions of the read.

Finally, we add a check to the beginning of Phase 1 to find an end-to-end alignment with no mismatches for the forward-oriented read, if one exists. In this way, we guarantee that alignments with no mismatches will always be preferred over alignments with one or more mismatches.

**Supplementary Figure 2. The four phases of the full Bowtie algorithm.**


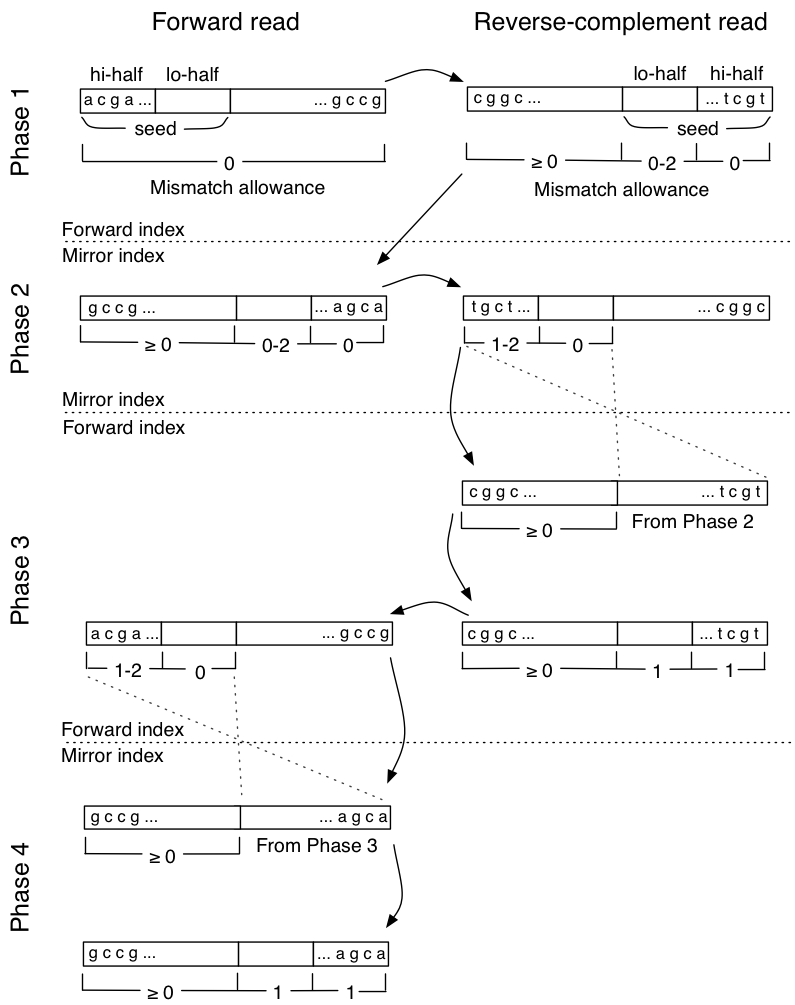


The full algorithm considers both the forward-oriented read and reverse-complement of the read. All four alignment “cases” are covered for both orientations.

**Supplementary Discussion 3:** Index Construction

Calculating a Burrows Wheeler Transform is closely related to building a suffix array.  Each element of the BWT is derivable from the corresponding element of the suffix array according to a formula:
**Supplementary equation 1**: **Relationship between BWT and suffix array**

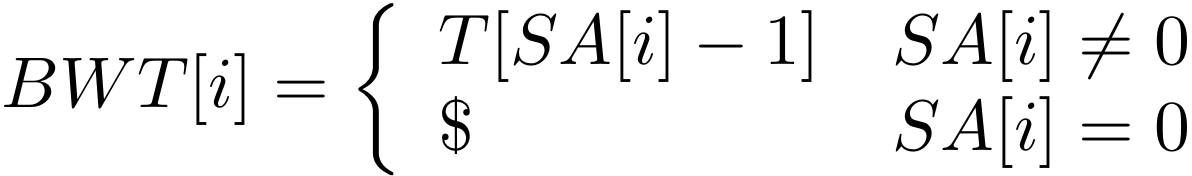


A conceptually simple way of calculating BWT is to build a suffix array using a suffix-sorting technique (e.g. Manber and Myers’ [2]), then calculate the BWT in a single pass over the suffix array. However, this technique has a very large memory footprint.  Constructing the entire suffix array in memory incurs a memory footprint of at least about 12 gigabytes for the human genome, for example.  This may be acceptable for users with access to large-memory computers, but our goal is to facilitate research on typical workstations.

Kärkkäinen [3] proposes a memory-conscious blockwise strategy.  His method builds the suffix array and the BWT block-by-block and in tandem, discarding suffix-array blocks once the corresponding BWT block has been built.  By setting a small block size relative to the length of the genome, the technique achieves a very small memory footprint.  The blockwise method also enables the user to trade flexibly between speed and peak memory usage by adjusting block size and other parameters. Bowtie's indexer adopts a form of Kärkkäinen's method and can build a full Bowtie index for the human genome in about 24 hours in less than 1.5 gigabytes of RAM.  If 16 gigabytes of RAM or more is available, the indexer can exploit the additional RAM to produce the same index in about 4.5 hours.
**Supplementary Discussion 4:** Index components

The largest single component of the Bowtie index is the BWT sequence.  Bowtie stores the BWT in a 2-bit-per-base format.  Inline character occurrence counts occupy about 14% the space of the packed BWT, and text offsets for marked rows occupy about 50% the space of the packed BWT.  With some other small structures, the overall Bowtie index for a given genome is about 65-70% larger than the packed BWT.  A Bowtie index for the assembled human genome sequence is about 1.3 gigabytes.  As mentioned, a full Bowtie index actually consists of pair of equal-size indexes, the forward and mirror indexes, for any given genome.  Bowtie can be run such that only one of the two indexes is ever resident in memory at once (using the –z option), so the memory footprint of Bowtie under those circumstances remains about 1.3 gigabytes. Without the –z option, the human index has a memory footprint of about 2.2 gigabytes, and 2.2 gigabytes is needed to store the index on disk in either case.

**Supplementary Table 1: Bowtie index building performance (full)**

| Physical memory  Target (gigabytes) | Actual peak memory footprint (gigabytes) | # suffix array blocks | Difference cover period | Bit-packed reference | Wall clock time |
| --- | --- | --- | --- | --- | --- |
| 16 | 14.4 | 1 | 256 | no | 4h:36m |
| 8 | 5.84 | 6 | 1024 | no | 5h:05m |
| 4 | 3.39 | 34 | 4096 | no | 7h:40m |
| 2 | 1.39 | 34 | 4096 | yes | 21h:30m |

Supplementary Table 1 presents memory footprints and wall clock times for a human-genome run of the indexer under parameters selected to satisfy different physical memory constraints. These runs were performed on a server with a 2.4 GHz AMD Opteron processor and 32 gigabytes of RAM. “Number of blocks” indicates how many blocks the blockwise algorithm used. “Difference cover period” indicates the periodicity of the up-front difference-cover-based pre-sort. This pre-sort, proposed by Burkhardt and Kärkkäinen [4], is a technique whereby a subset of the suffixes are pre-sorted and then used to avoid quadratic runtimes in downstream stages of the indexer. Shorter periods require more up-front work to calculate the pre-sort and more memory to store it, but also yield shorter running times in downstream stages. “2-bit-per-base references” indicates whether a bit-packed representation of the reference sequence was used. The bit-packed representation reduces memory footprint but increases running time.

References

1. Ferragina P, Manzini G: **Opportunistic data structures with applications**. In: *Proceedings of the 41st Annual Symposium on Foundations of Computer Science.* IEEE Computer Society; 2000.

2. Manber U, Myers G: **Suffix arrays: a new method for on-line string searches**. In: *Proceedings of the first annual ACM-SIAM symposium on Discrete algorithms.* San Francisco, California, United States: Society for Industrial and Applied Mathematics; 1990.

3. Kärkkäinen J: **Fast BWT in small space by blockwise suffix sorting**. *Theor Comput Sci* 2007, **387**(3):249-257.

4. Burkhardt S, Kärkkäinen J: **Fast lightweight suffix array construction and checking**. In: *14th Annual Symposium on Combinatorial Pattern Matching: 2003; Morelia, Michoacn, Mexico*; 2003.
